# Presence, Subtypes, and Prognostic Significance of Tertiary Lymphoid Structures in Urothelial Carcinoma of the Bladder

**DOI:** 10.1093/oncolo/oyad283

**Published:** 2023-10-24

**Authors:** Guofeng Ma, Huiqing Jia, Guofang Zhang, Ye Liang, Xianning Dong, Guangming Fu, Xinsheng Wang, Haitao Niu

**Affiliations:** Department of Urology, The Affiliated Hospital of Qingdao University, Qingdao, People’s Republic of China; Department of Pathology, The Affiliated Hospital of Qingdao University, Qingdao, People’s Republic of China; Department of Gynecology, The Affiliated Hospital of Qingdao University, Qingdao, People’s Republic of China; Key Laboratory, Department of Urology and Andrology, The Affiliated Hospital of Qingdao University, Qingdao, People’s Republic of China; Department of Pathology, The Affiliated Hospital of Qingdao University, Qingdao, People’s Republic of China; Department of Pathology, The Affiliated Hospital of Qingdao University, Qingdao, People’s Republic of China; Department of Urology, The Affiliated Hospital of Qingdao University, Qingdao, People’s Republic of China; Department of Urology, The Affiliated Hospital of Qingdao University, Qingdao, People’s Republic of China

**Keywords:** tertiary lymphatic structures, tumor-infiltrating lymphocytes, urothelial carcinoma of the bladder, NMIBC, MIBC, prognosis

## Abstract

**Objective:**

To evaluate the presence and subtypes of tertiary lymphatic structures (TLSs) in urothelial carcinoma of the bladder (UCB) and to analyze their associated clinicopathological characteristics and prognostic significance.

**Methods:**

The study enrolled 580 patients with surgically treated UCB, including 313 non-muscle invasive bladder cancer (NMIBC) and 267 muscle-invasive bladder cancer (MIBC). The presence and subtypes of TLSs were identified by immunohistochemistry (CD20, CD3, Bcl-6, and CD21). TLSs were classified into non-GC (nGC) TLS and GC TLS subtypes based on germinal center (GC) formation. Disease-free survival (DFS) was used as an endpoint outcome to evaluate the prognostic significance of TLS and its subtypes in UCB.

**Results:**

TLSs were more common in MIBC than in NMIBC (67.8% vs 48.2%, *P* < .001), and the tumor-infiltrating lymphocyte (TIL) mean density was significantly higher in MIBC than in NMIBC (24.0% vs 17.5%, *P* < .001). Moreover, a positive correlation was found between TLS presence and GC structure formation and TIL infiltration in UCB. Endpoint events occurred in 191 patients. Compared to patients with endpoint events, patients without disease progression exhibited higher TIL density and more TLSs (*P* < .05). Kaplan–Meier curves showed that TLS was associated with better DFS in NMIBC (*P* = .041) and MIBC (*P* = .049). However, the Cox multivariate analysis did not demonstrate the prognostic significance of TLS.

**Conclusions:**

TLS is heterogeneous in UCB, and that TLS and GC structures are related to TIL density and prognostic events. However, TLS as a prognostic indicator remains unclear, warranting further investigation.

## Teaser

Urothelial carcinoma of the bladder (UCB) is a common malignant tumor in the urinary system. This article evaluates the presence and subtypes of tertiary lymphatic structures (TLSs) in a large number of UCB clinical samples and analyzes their associated clinicopathological characteristics and prognostic significance.

**Implications for Practice**

The researchers used well-established pathological techniques, including hematoxylin and eosin (H&E) staining and immunohistochemistry (IHC), as well as standardized, clinically applicable, and highly reproducible assessments, to evaluate the presence, subtype/maturity, and prognostic significance of tertiary lymphatic structures (TLSs) in a large number of urothelial carcinomas of the bladder (UCB) clinical samples. The study revealed crucial TLS features in UCB and explored the clinical feasibility of TLS as a prognostic biomarker for patients with UCB. These findings could have implications for clinical practice and further TLS research.

## Introduction

Urothelial carcinoma of the bladder (UCB) is a common malignant tumor in the urinary system. It is classified into non-muscle invasive bladder cancer (NMIBC) and muscle-invasive bladder cancer (MIBC). The 5-year progression rate of high-risk NMIBC is up to 40% and transurethral resection of bladder tumor (TURBT) combined with intravesical instillation, depending on the risk classification, is its standard treatment approach.^1,2^ On the other hand, radical cystectomy (RCT) is the recommended treatment option for MIBC, which has a poor prognosis with a 60%-70% 5-year overall survival (OS) rate.^3^ In recent years, immunotherapy has emerged as a promising treatment alternative for patients with metastatic UCB. However, only a few patients have a long-term, durable response to immunotherapy.^3^ Consequently, the central focus of UCB research has shifted to identifying novel biomarkers that can accurately stratify prognosis, manage treatment, and predict the response to or efficacy of immunotherapy.

TLSs are ectopic lymphocyte aggregates formed in nonlymphocytic tissues that can develop at sites or target organs of inflammation, autoimmune diseases, and tumors. They present all the features of lymph node structures associated with the generation of an adaptive immune response, including a T-cell zone with mature dendritic cells (DCs), a germinal center (GC) with follicular dendritic cells (FDCs) and proliferating B cells, and high endothelial venules (HEVs).^4^ Recent studies reported that TLSs correlate with improved clinical outcomes and immunotherapy responses in most tumor types,^5^ with a few exceptions.^6^ However, studies on TLSs in UCB are constrained by small sample sizes.^7-9^ Consequently, the presence and characteristics of TLSs, as well as their clinical evaluation and prognostic significance, remain unclear.

Herein, TLSs were divided into the nGC TLS and GC TLS subtypes based on GC formation (a critical TLS maturation characteristic) for the practicality of this clinicopathological investigation. Additionally, we evaluated the TILs, which are significant constituents of the immune microenvironment, encompassing T cells, B cells, natural killer cells, and macrophages. Few studies have associated TILs^9^ and their important cell subset CD8^+^T cells^10,11^ with better survival outcomes in UCB. We aimed to evaluate the presence and subtypes of TLSs in 580 patients with UCB, as well as their clinicopathological features and prognostic value.

## Materials and Methods

### Collection of Clinical Samples and Data

This retrospective study was ethically approved by the Ethics Committee of the Affiliated Hospital of Qingdao University (approval No.: QYFY WZLL 27571), China. It included 580 patients with UCB recruited between January 2017 and December 2019. All patients underwent surgical treatment. The exclusion criteria were as follows: (1) Patients with a concurrent presence of other malignant tumors; (2) Patients who have undergone preoperative immunotherapy, targeted therapy, or neoadjuvant therapy; and (3) Insufficient surgical specimens for subsequent immunohistochemical (IHC) testing. Patients’ clinicopathological data (gender, age, tumor size, tumor multiplicity, histological subtype, T stage, lymph node metastasis, nerve invasion, lymphovascular invasion (LVI), Ki67 expression, treatment method, and surgical resection status) were obtained from a medical record system and a pathological database. Two uropathologists systematically reviewed and verified all cases based on the eighth edition International Union against Cancer (UICC) TNM Classification of Malignant Tumors^12^ and the 2016 World Health Organization Urogenital Tumor Classification.^13^ Disease-free survival (DFS), the period between surgery and the occurrence of the first disease progression event (recurrence or metastasis) or death was used as the study endpoint. Patient follow-up was conducted through the clinical review of records or by telephone, and the last follow-up was scheduled for December 25, 2022.

### Immunohistochemistry (IHC)

Postoperative formalin-fixed paraffin-embedded tumor tissue samples were obtained from patients then sectioned into 4 μm thick slices. The slices were then subjected to deparaffinization in xylene, hydration, and antigen retrieval using Tris–EDTA (pH 9.0). A 3% hydrogen peroxide solution was used to inactivate the endogenous peroxidase. The sections were then incubated overnight at 4 °C with the following primary antibodies: CD20 (kit-0001, mouse monoclonal, MXB Biotec, 1:150); CD3 (MAB-0740, mouse monoclonal, MXB Biotec, 1:150); Bcl-6 (MAB-0746, mouse monoclonal, MXB Biotec, 1:100); and CD21 (MAB-0708, mouse monoclonal, MXB Biotec, 1:200). Following that, the sections were incubated with secondary antibodies for 40 minutes at 37 °C, and 3,3-diaminobenzidine (DAB) was used as the chromogen. Finally, the slices were counterstained with hematoxylin to visualize the nuclei, and CD20, CD3, and CD21 expressions were localized to the cell membrane, whereas the Bcl-6 expression was localized to the nucleus.

### Assessment of TLSs and TILs

**Figure 1.**
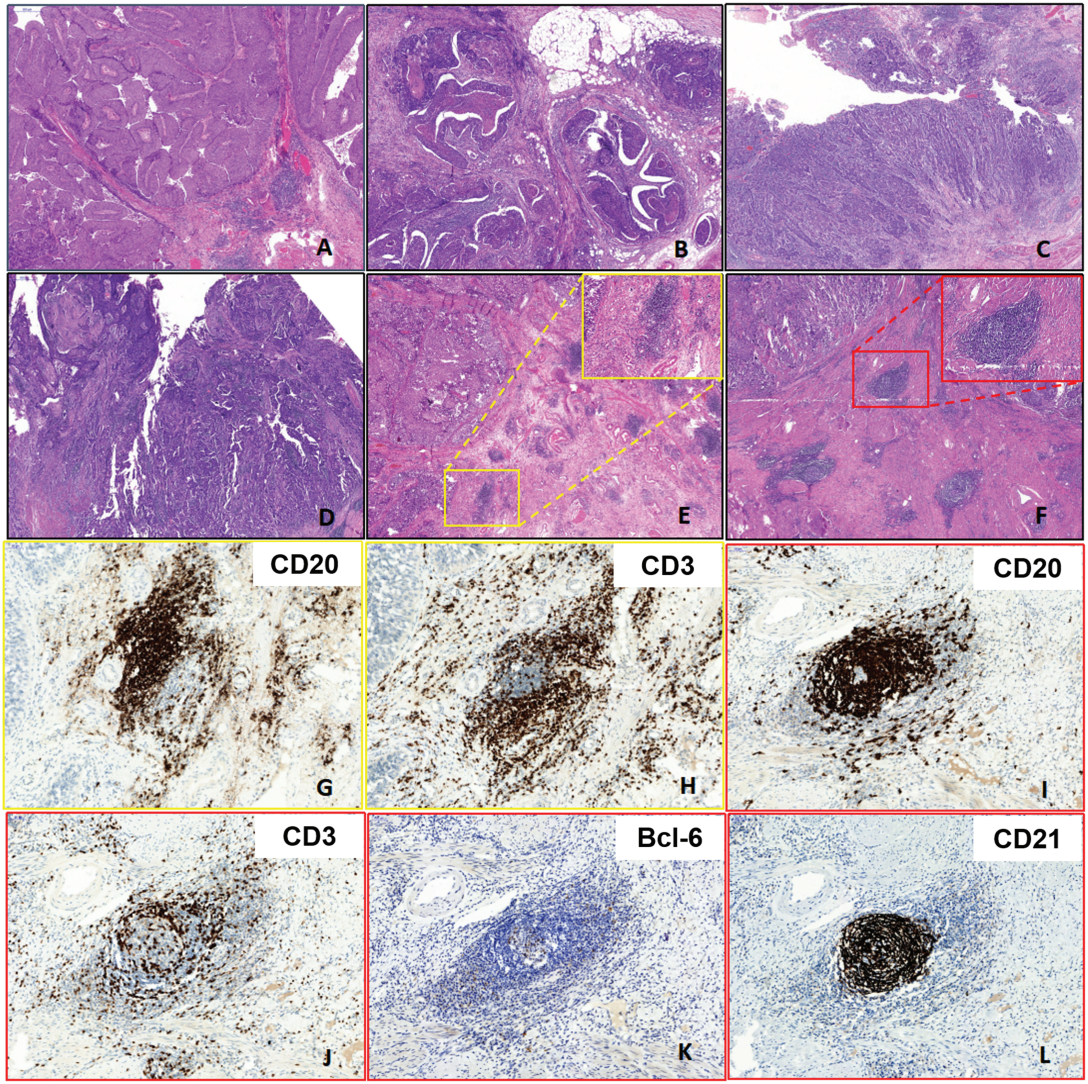
Representative hematoxylin and eosin (H&E) images of different tumor-infiltrating lymphocytes (TILs) density and tertiary lymphoid structures (TLSs) subtypes. (**A**: TILs = 20%; **B**: TILs = 40%; **C**: TILs = 60%; **D**: TILs = 80%; **E:** nGC TLSs; **F**: GC TLSs; A-F: 40x; E and F Insert:200×). Representative immunohistochemistry (IHC) images of non-germinal center (nGC) TLS and germinal center (GC) TLS (nGC TLS: **G**: CD20 + B cells; **H**: CD3 + T cells; GC TLS: **I**: CD20 + B cells; **J**: CD3 + T cells; **K**: Bcl-6 + GC B cells; **L**: CD21 + FDCs; 200×).

Our analysis was focused on determining the presence and identifying the subtypes of TLSs. We first observed round or round-like lymphocyte aggregates in the hematoxylin and eosin (H&E) sections within the tumor stroma, and peritumoral and intratumoral tissues. Second, we used IHC to establish the presence of TLSs by combining the expression of CD20^+^ B cells and CD3^+^ T cells. The presence of TLS structures was not considered when only B cells or T cells aggregated without forming lymphoid aggregates. Finally, TLSs were subdivided into nGC TLS or GC TLS subtypes based on GC formation. Typical well-developed GC structures were observed on the H&E sections, and using the Bcl-6 and CD21 markers for identifying ambiguous GC structures proved beneficial. While the nGC TLSs (Fig. 1E, 1G and 1H) were characterized by CD20^+^ B cell structures surrounded by CD3^+^ T cells without GCs, the GC TLSs (Fig. 1F, 1I-1L) were defined by the presence of GC structures within lymphoid aggregates characterized by Bcl-6^+^ GC B cells and CD21^+^ FDCs.

Here, stromal TILs in UCB were manually evaluated using H&E sections based on the standardized methodology proposed by the International Immuno-Oncology Biomarkers Working Group for assessing TILs in solid tumors^14^ which is detailed as follows. First, when assessing TILs in UCB, the TIL assessment area should be determined within the borders of the invasive tumor, which includes the invasive edge and the stroma pertaining to the fibrovascular cores of invasive papillary structures. At the same time, necrosis, coagulavon artefacts, and the previous biopsy area should be excluded. Subsequently, the stromal TILs (located within the tumor stroma) in the aforementioned areas should be evaluated, excluding intratumoral TILs (found within the tumor epithelial cancer nests). Notably, TILs encompass all mononuclear leukocytes, such as lymphocytes and plasma cells, and exclude polymorphonuclear leukocytes and eosinophils. Moreover, TIL density is defined as the percentage of stromal TIL area in the tumor stromal region. The illustrative tutorial detailing the standard approach for assessing TILs in urothelial carcinoma can be found in the “Supplementary_Data_File_6” of the article published by the International Immuno-Oncology Biomarkers Working Group (https://www.ncbi.nlm.nih.gov/pmc/articles/PMC5638696/).^14^ The UCB images with different TIL densities are depicted in Fig. 1A-1D. Herein, two pathologists completed the assessment of TLSs and TILs and reached a consensus regarding their findings.

### Statistical Analysis

The chi-square test or Fisher’s exact test was used to analyze categorical variables, while continuous variables were analyzed using the *t*-test or one-way analysis of variance (ANOVA). Survival curves were plotted and compared using the Kaplan–Meier method and the log-rank test, respectively. A multivariate Cox regression model was employed to evaluate the prognostic significance of TLSs and TILs, controlling for all relevant clinicopathological covariates. All statistical tests were 2-sided, and results with *P* < .05 were considered statistically significant. Data processing and plotting were performed using IBM SPSS V.26.0 or GraphPad Prism V.8.1.

## Results

### Clinicopathologic Characteristics of Patients

**Figure 2.**
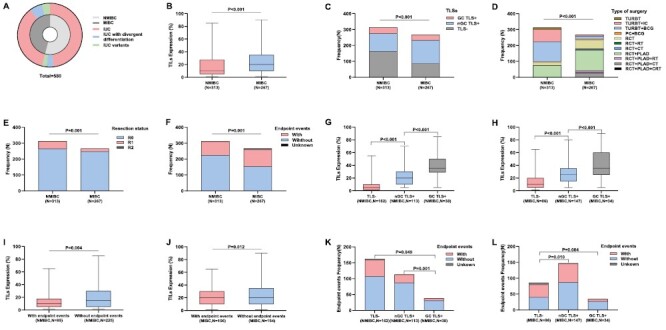
**(A**) Pie chart of histological subtypes for non-muscle invasive bladder cancer (MIBC) and patients with muscle-invasive bladder cancer (MIBC) (IUC: invasive urothelial carcinoma). (**B**) Tumor-infiltrating lymphocytes (TILs) expression in NMIBC and MIBC (*P* <.001, *t* test). (**C**) Tertiary lymphoid structures (TLSs) in NMIBC and MIBC (*P* <.001, chi-square test). (**D**) Type of surgery in NMIBC and MIBC (*P* = .001, chi-square test). (**E**) Resection status in NMIBC and MIBC (*P* = .001, chi-square test). (**F**) Endpoint events in NMIBC and MIBC (*P* = .001, Chi-square test). (**G**) TILSs expression according to TLSs in NMIBC (TLS- vs nGC TLS: *P* <.001; TLS- vs GC TLS: *P* <.001; nGC TLS vs GC TLS: *P* <.001; one-way ANOVA, multiple comparisons). (**H**) TILSs expression according to TLSs in MIBC (TLS- vs nGC TLS: *P* <.001; TLS- vs GC TLS: *P* <.001; nGC TLS vs GC TLS: *P* <.001; one-way ANOVA, multiple comparisons). (**I**) Expression of TILs in patients with NMIBC with or without endpoint events (*P* = .004, *t* test). (**J**) Expression of TILs in patients with MIBC with or without endpoint events (*P* = 0.012, *t* test). (**K**) TLSs in patients with NMIBC with or without endpoint events (TLS- vs nGC TLS: *P* = .076; TLS- vs GC TLS: *P* = .049; nGC TLS vs GC TLS: *P* = .001; chi-square test). (**L**) TLSs in patients with MIBC with or without endpoint events (TLS- vs nGC TLS: *P* = .010; TLS- vs GC TLS: *P* = .004; nGC TLS vs GC TLS: *P* = .085; chi-square test).

Among 580 patients, 480 (82.8%) were male and 100 (17.2%) were female, with a male-to-female ratio of 4.8:1. The median age and tumor diameter were 68 (30-93) years and 3 (0.2-15) cm, respectively. There were 313 (54.0%) patients with NMIBC and 267 (46.0%) patients with MIBC. All patients’ tumor histological types were invasive urothelial carcinoma (IUC), including less common subtypes such as IUC with divergent differentiation (NMIBC: 21/313; MIBC: 37/267) and IUC variants (NMIBC: 13/313; MIBC: 23/267) (Fig. 2A). Regarding treatment procedures, 68.7% (215/313) of patients with NMIBC underwent TURBT, while 48.3% (129/267) of patients with MIBC underwent RCT with pelvic lymph node dissection. Supplementary Table S1 displays the detailed clinicopathological data of the patients. The median follow-up period was 45.7 (1-71) months. Median survival time for patients with NMIBC and MIBC was 42.8 and 36.5 months, respectively. The 3-year DFS rates for patients with NMIBC and MIBC were 77.3% and 62.9%, respectively. A total of 191 patients (32.9%) experienced an endpoint event, 85 in the NMIBC group and 106 in the MIBC group. Among these events, 93 patients (16.0%) died during follow-up, including 31 in the NMIBC group and 62 in the MIBC group. Furthermore, 10 patients were lost to follow up.

### Characteristics of TLSs and TILs in the NMIBC and MIBC Patient Groups

TLSs often develop in the peritumoral area as round or round-like lymphocyte aggregates, easily noticeable in H&E sections, and are rarely found in the intratumoral region (within tumor epithelial cancer nests). Only 3 MIBC cases had intratumoral TLSs in H&E (not confirmed by IHC due to insufficient tissue), and all 3 cases exhibited clear GC TLS at the peritumoral location.

Our analysis revealed that TLSs were present in 48.2% (151/313) and 67.8% (181/267) of NMIBC and MIBC cases, respectively (Fig. 2C). Furthermore, GC structures were uncommon among patients with TLSs, occurring in 38 (25.2%) and 34 (18.8%) NMIBC and MIBC cases, respectively. Table 1 shows a comparison between TLSs and TILs in patients with NMIBC and MIBC. Specifically, patients with MIBC had more TLSs (*P* < .001) and a higher TIL density (*P* < .001, Fig. 2B) than patients with NMIBC. Regarding TLS subtypes, the proportion of nGC TLS was significantly higher in patients with MIBC than in patients with NMIBC (55.1% vs 36.1%; *P* < .001), and the 2 patient groups showed no significant difference in GC TLS (12.7% vs 12.1%; *P* = .829). Additionally, there were significant differences in treatment modality (*P* < .001, Fig. 2D), surgical resection status (*P* = .001, Fig. 2E), and endpoint events (*P* = .001, Fig. 2F) between the NMIBC and MIBC patient groups.

**Table 1.** Comparison of TILs density and TLSs in NMIBC and MIBC.

Clinicopathological	All	NMIBC	MIBC	*P*
**Characteristics**	(580, 100%)	(313, 54.0%)	(267, 46.0%)	
**TLSs**				
Positive	332(57.2)	151(48.2)	181(67.8)	**<.001**
Negative	248(42.8)	162(51.8)	86(32.2)	
**nGC TLS**				
Positive	260(44.8)	113(36.1)	147(55.1)	**<.001**
Negative	320(55.2)	200(63.9)	120(44.9)	
**GC TLS**				
Positive	72(12.4)	38(12.1)	34(12.7)	.829
Negative	508(87.6)	275(87.9)	233(87.3)	
**TILs**				
Median/range	15/1-90	10/1-85	20/1-90	**<.001** ^a^
Mean ± SD	20.5 ± 17.0	17.5 ± 16.2	24.0 ± 17.2	

*P* value bolded: *P* < 0.05.

^a^: *t*-test; other testing methods are chi-square test.

Abbreviations: GC TLS: germinal center TLS; MIBC: muscle-invasive bladder cancer; nGC TLS: non-germinal center TLS; NMIBC: non-muscle invasive bladder cancer; TILs: tumor-infiltrating lymphocytes; TLSs: tertiary lymphoid structures.

### Comparison of the Clinicopathological Characteristics of Groups Classified Based on TLS Presence and Subtype

We further compared the correlation between clinicopathological features in both the NMIBC (Table 2) and MIBC (Table 3) patient groups using TLS presence (TLSs + vs TLSs-) and subtypes (nGC TLS vs GC TLS+) as the grouping variables. Interestingly, the TIL density was higher in the TLSs+ group than in the TLSs-group in both the NMIBC and MIBC patient groups (NMIBC: *P* < .001; MIBC: *P* < .001), with fewer endpoint events in the TLSs+ group (NMIBC: *P* = .032; MIBC: *P* = .043). Furthermore, the TIL density was higher in the GC TLS group than in the nGC TLS group (NMIBC: *P* < .001; MIBC: *P* < .001). Figure 2G and 2H depicts the relationship between TLSs and TIL density in the NMIBC and MIBC groups, showing a positive correlation between TLS formation and GC structures with TIL infiltration. Additionally, the results indicate that the TLSs+ group had a higher LVI than the TLSs-group (*P* = .020) in the NMIBC patient group, while the TLSs+ group had smaller tumors compared to the TLSs-group (*P* = .002), in the MIBC patient group.

**Table 2.** Clinicopathological characteristics comparison according to the presence and subtypes of TLSs in NMIBC.

Clinicopathological	TLSs+	TLSs-	*P*	nGC TLS+	GC TLS+	*P*
Characteristics	(*N* = 151)	(*N* = 162)		(*N* = 113)	(*N* = 38)	
**Age/years**						
Median/range	68/38-94	68/30-90	.669^a^	69/41-94	66.5/38-91	.545^a^
Mean ± SD	67.9 ± 10.2	68.4 ± 10.0		68.2 ± 9.9	67.0 ± 10.9	
**Gender**						
Male	123(81.5)	130(80.2)	.786	92(81.4)	31(81.6)	.982
Female	28(18.5)	32(19.8)		21(18.6)	7(18.4)	
**Tumor size/cm**						
Median/range	3/0.2-8	3/0.3-10	.733^a^	3/0.2-8	3/0.5-7	.132^a^
Mean ± SD	3.0 ± 1.7	3.0 ± 1.8		2.9 ± 1.8	3.4 ± 1.4	
**Tumor multiplicity**						
Single	121(80.1)	140(86.4)	.135	88(77.9)	33(86.8)	.231
Multiple	30(19.9)	22(13.6)		25(22.1)	5(13.2)	
**Histological subtype**						
IUC	135(89.4)	144(88.8)	.323	101(89.4)	34(89.5)	1.000
IUC with divergent differentiation	12(7.9)	9(5.6)		9(8.0)	3(7.9)	
IUC variants	4(2.7)	9(5.6)		3(2.6)	1(2.6)	
**Lymph node metastasis**						
Positive	2(1.3)	1(0.6)	.898	2(1.8)	10(26.3)	1.000
Negative	46(30.5)	27(16.7)		36(31.9)	0	
Unknown	103(68.2)	134(82.7)		75(66.3)	28(73.7)	
**Perineural Invasion**						
Positive	1(0.7)	5(3.1)	.118	1(0.9)	38(100.0)	1.000
Negative	150(99.3)	157(96.9)		112(99.1)	0	
**LVI**						
Positive	11(7.3)	3(1.9)	**.020**	7(6.2)	4(10.5)	.597
Negative	140(92.7)	159(98.1)		106(93.8)	34(89.5)	
**TILs**						
Median/range	25/5-85	5/1-55	**<.001** ^*^	20/5-70	40/10-60	**<.001** ^*^
Mean ± SD	26.9 ± 16.7	8.8 ± 9.7		22.9 ± 14.0	36.7 ± 15.8	
**Ki-67**						
Median/range	40/2-80	30/1-90	.171^*^	40/2-80	35/5-85	.609^*^
Mean ± SD	39.8 ± 20.2	33.4 ± 25.7		40.5 ± 21.1	38.7 ± 18.4	
Unknown	99	115		70	29	
**Treatment method**						
TURBT	4(2.6)	7(4.3)	**.014**	3(2.7)	1(2.6)	.503
TURBT + IC	29(19.2)	49(30.2)		24(21.2)	5(13.2)	
TURBT + BCG	56(37.1)	70(43.2)		37(32.7)	19(50.0)	
PC + BCG	2(1.3)	2(1.3)		2(1.8)	0	
RCT	13(8.6)	7(4.3)		10(8.9)	3(7.9)	
RCT + PLND	47(31.1)	27(16.7)		37(32.7)	10(26.3)	
**Resection status**						
R0	130(86.1)	134(82.7)	.411	99(87.6)	31(81.6)	.353
R1	21(13.9)	28(17.3)		14(12.4)	7(18.4)	
R2	0	0		0	0	
**Prognostic events**						
With	33(21.9)	52(32.1)	**.032**	26(23.0)	7(18.4)	.554
Without	118(78.1)	107(66.0)		87(77.0)	31(81.6)	
Unknown	0	3(1.9)		0	0	

*P* value bolded: *P* < .05.

^a^ *t*-Test; other testing methods are chi-square test.

Abbreviations: BCG: BCG intravesical immunotherapy; GC TLS: germinal center TLSs; IC: intravesical chemotherapy; IUC: Invasive urothelial carcinoma; LVI: lymphovascular invasion; nGC TLS: non-germinal center TLSs; NMIBC: non-muscle invasive bladder cancer; PC: partial cystectomy; PLND: pelvic lymph node dissection; RCT: radical cystectomy; TILs: tumor-infiltrating lymphocytes; TLSs: tertiary lymphoid structures; TURBT: transurethral resection of bladder tumor.

**Table 3.** Clinicopathological characteristics comparison according to the presence and subtypes of TLSs in MIBC.

Clinicopathological	TLSs+	TLSs-	*P*	nGC TLS+	GC TLS+	*P*
characteristics	(*N* = 181)	(*N* = 86)		(*N* = 147)	(*N* = 34)	
**Age/years**						
Median/range	68/45-92	68.5/36-93	.571^a^	68/45-92	67/51-91	.972^a^
Mean ± SD	68.1 ± 10.0	68.8 ± 11.0		68.1 ± 10.4	68 ± 8.7	
**Gender**						
Male	155(85.6)	72(83.7)	.682	127(86.4)	28(82.4)	.545
Female	26(14.4)	14(16.3)		20(13.6)	6(17.6)	
**Tumor size/cm**						
Median/range	3.5/1-10	4.5/1-15	**.002** ^a^	4/1-10	3.5/1.7-7	.137^a^
Mean ± SD	3.9 ± 1.7	4.9 ± 2.6		4.0 ± 1.8	3.5 ± 1.4	
**Tumor multiplicity**						
Single	152(84.0)	77(89.5)	.225	123(83.7)	29(85.3)	.816
Multiple	29(16.0)	9(10.5)		24(16.3)	5(14.7)	
**Histological subtype**						
IUC	146(80.7)	61(70.9)	.199	118(80.2)	28(82.4)	.525
IUC with divergent differentiation	22(12.2)	15(17.5)		17(11.6)	5(14.7)	
IUC variants	13(7.1)	10(11.6)		12(8.2)	1(2.9)	
**Lymph node metastasis**						
Positive	34(18.8)	15(17.4)	.809	24(16.3)	10(29.4)	**.036**
Negative	83(45.9)	40(46.5)		72(49.0)	11(32.4)	
Unknown	64(35.3)	31(36.1)		51(34.7)	13(38.2)	
**pT**						
2	101(55.8)	41(47.7)	.460	80(54.4)	121(51.9)	.738
3	61(33.7)	34(39.5)		51(34.7)	85(36.5)	
4	19(10.5)	11(12.8)		16(10.9)	27(11.6)	
**Perineural Invasion**						
Positive	47(26.0)	27(31.4)	.354	38(25.9)	9(26.5)	.941
Negative	134(74.0)	59(68.6)		109(74.1)	25(73.5)	
**LVI**						
Positive	68(37.6)	33(38.4)	.899	52(35.4)	16(47.1)	.205
Negative	113(62.4)	53(61.6)		95(64.6)	18(52.9)	
**TILs**						
Median/range	25/5-90	10/1-65	**<.001** ^a^	25/5-80	35/5-90	**<.001** ^a^
Mean ± SD	29.2 ± 17.0	13.0 ± 11.6		26.4 ± 14.6	41.2 ± 21.3	
**Ki67**						
Median/range	25/5-90	40/4-85	.073	40/5-90	60/15-80	.062
Mean ± SD	29.2 ± 17.0	41.8 ± 22.4		46.3 ± 21.1	56.1 ± 17.4	
Unknown	0	27		52	15	
**Treatment method**						
TURBT + IC	7(3.9)	1(1.2)	.345	6(4.1)	1(2.9)	.716
TURBT + BCG	8(4.4)	10(11.6)		8(5.5)	0	
PC + BCG	4(2.2)	2(2.3)		4(2.7)	0	
RCT	39(21.5)	16(18.6)		28(19.0)	11(32.4)	
RCT + RT	2(1.1)	1(1.2)		2(1.4)	0	
RCT + CT	4(2.2)	2(2.3)		3(2.0)	1(2.9)	
RCT + PLND	91(50.3)	38(44.2)		75(51.0)	16(47.1)	
RCT + PLND + RT	6(3.3)	6(7.0)		5(3.4)	1(2.9)	
RCT + PLND + CT	17(9.4)	10(11.6)		14(9.5)	3(8.9)	
RCT + PLND + CRT	3(1.7)	0		2(1.4)	1(2.9)	
**Resection status**						
R0	171(94.5)	77(89.5)	.108	138(93.9)	33(97.1)	.705
R1	8(4.4)	9(10.5)		7(4.8)	1(2.9)	
R2	2(1.1)	0		2(1.3)	0	
**Prognostic events**						
With	66(36.5)	40(46.5)	**.043**	59(40.1)	7(20.6)	**.033**
Without	114(63.0)	40(46.5)		87(59.2)	27(79.4)	
Unknown	1(0.5)	6(7.0)		1(0.7)	0	

*P* value bolded: *P* < .05.

^a^ *t*-Test; other testing methods are chi-square test.

Abbreviations: BCG: BCG intravesical immunotherapy; CRT: chemoradiotherapy; CT: chemotherapy; GC TLSs: germinal center TLSs; IC: intravesical chemotherapy; IUC: invasive urothelial carcinoma; LVI: lymphovascular invasion; MIBC: muscle-invasive bladder cancer; nGC TLSs: non-germinal center TLSs; PC: partial cystectomy; PLND: pelvic lymph node dissection; RCT: radical cystectomy; RT: radiotherapy; TILs: tumor-infiltrating lymphocytes; TLSs: tertiary lymphoid structures; TURBT: transurethral resection of bladder tumor.

### Prognostic Significance of TILs and TLSs

Finally, we obtained the prognostic information of 570 patients and analyzed the relationship between endpoint events, TLS presence, and TIL density in the NMIBC and MIBC patient groups. According to the results, patients without disease progression had a higher TIL density than those with endpoint events in both the NMIBC (*P* = .004, Fig. 2I) and MIBC (*P* = .012, Fig. 2J) patient groups. Furthermore, compared to patients with TLSs, patients with TLSs+ had significantly fewer endpoint events (NMIBC: *P* = 0.032; MIBC: *P* = .043), and patients with GC TLS had the lowest rate of endpoint events (NMIBC: 7/38; MIBC: 7/34; Fig. 2K-2L).

**Figure 3.**
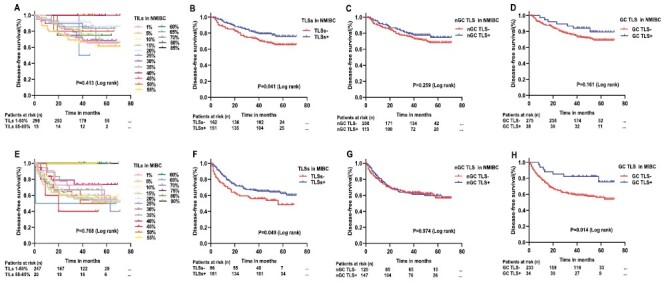
Kaplan–Meier curves of disease-free survival (DFS) for non-muscle invasive bladder cancer (NMIBC) according to TILs density (**A**, *P* = .413,), TLSs (**B**, *P* = .041), nGC TLS (**C**, *P* = .259), and GC TLS (**D**, *P* = .161). Kaplan–Meier curves of DFS for muscle-invasive bladder cancer (MIBC) according to TILs density (**E**, *P* = .768), TLSs (**F**, *P* = .049), nGC TLS (**G**, *P* = .974), and GC TLS (**H**, *P* = .014).

Kaplan–Meier curves (Fig. 3) showed that NMIBC (*P* = .041) and MIBC (*P* = 0.049) patients with TLSs had better DFS, patients with MIBC with GC TLS showing a more favorable DFS (*P* = .014). Survival curves showed no statistical difference (NMIBC: *P* = .413; MIBC: *P* = .768) based on TIL density (continuous score). The Cox multivariate analysis revealed that lymph node metastasis (hazard ratio [HR]: 72.009, 95% confidence interval [CI]: 10.046-516.142, *P* < .001) was an independent factor affecting DFS in patients with NMIBC. On the other hand, age (HR: 1.037, 95% CI: 1.004-1.072, *P* = .027), histological subtype (HR: 2.283, 95% CI: 1.220-4.274, *P* = .010), and Ki-67 (HR: 1.018, 95% CI: 1.002-1.035, *P* = .031) were identified as independent prognostic factors for patients with MIBC. However, our analysis could not confirm the prognostic significance of TILs and TLSs (Table 4).

**Table 4.** Univariate and multivariate Cox regression analyses of DFS in patients with NMIBC and MIBC.

	Univariate analysis		Multivariate model^a^	
NMIBC	HR (95% CI)	*P*	HR (95% CI)	*P*
**Age/years** 30-90	1.030(1.007-1.054)	**.010**	1.074(0.976-1.182)	.146
**Histological subtype** IUC vs others	2.922(1.752-4.873)	**<.001**	1.502(0.014-2.181)	.705
**Histological grade** low vs high	2.807(1.136-6.936)	**.025**	1.543(0.182-13.072)	.691
**Lymph node metastasis** negative vs positive	28.560(6.213-131.297)	**<.001**	72.009(10.046-516.142)	**<.001**
**TILs** 1-85%	0.977(0.961-0.993)	**.004**	0.982(0.935-1.031)	.463
**TLSs** negative vs positive	0.638(0.413-0.987)	**.044**	0.619(0.123-3.127)	.562
**Type of surgery** non-RCT vs RCT	0.413(0.233-0.733)	**.002**	0.172(0.014-2.181)	.174
**MIBC**				
**Age/years** 36-93	1.026(1.006-1.046)	**.001**	1.037(1.004-1.072)	**.027**
**Tumor multiplicity** single vs multiple	2.056(1.038-4.072)	**.039**	1.505(0.608-3.726)	.377
**Histological subtype** IUC vs others	2.949(1.995-4.360)	**<.001**	2.283(1.220-4.274)	**.010**
**Lymph node metastasis** negative vs positive	2.545(1.560-4.152)	**<.001**	1.024(0.477-2.202)	.951
**pT** T2 vs T3-4	2.442(1.643-3.630)	**<.001**	2.218(0.951-5.174)	.065
**Perineural invasion** negative vs positive	1.833(1.237-2.716)	**.003**	1.229(0.630-2.401)	.545
**LVI** negative vs positive	2.097(1.431-3.073)	**<.001**	1.371(0.667-2.820)	.391
**TILs** 1-90%	0.985(0.973-0.997)	**.015**	0.986(0.963-1.010)	.264
**GC TLSs** negative vs positive	0.398(0.185-0.858)	**.019**	0.231(0.037-1.428)	.115
**Ki-67** 4-90%	1.015(1.003-1.026)	**.011**	1.018(1.002-1.035)	**.031**
**Type of surgery** non-RCT vs RCT	1.714(1.043-2.818)	**.034**	1.676(0.518-5.423)	.389
**Resection status** R0 VS R1-2	2.570(1.461-4.521)	**.001**	3.493(0.999-12.218)	.050

*P* value bolded: *P* < .05.

^*^Perform univariate analysis on all clinicopathological and include significant variables (*P* < .05) in multivariable Cox regression analysis.

Abbreviations: GC TLS: germinal center TLSs; IUC: Invasive urothelial carcinoma; LVI: lymphovascular invasion; MIBC: muscle-invasive bladder cancer; NMIBC: non-muscle invasive bladder cancer; RCT: radical cystectomy; TILs: tumor-infiltrating lymphocytes; TLSs: tertiary lymphoid structures.

## Discussion

Under pathological conditions, the presence of persistent antigenic stimulation leads to lymphocyte accumulation in nonlymphoid tissues, where they initiate lymphoid neogenesis. The lymphoid neogenesis sometimes leads to TLS formation, which can be found in infections,^15^ autoimmune diseases,^16^ and malignancies^17^ and is generally considered the hallmark of an active immune response. The presence of TLSs in tumors varies with different differentiation stages, reflecting the various stages of lymphoid neogenesis. Specifically, the TLS development spectrum ranges from simple lymphocyte aggregate clusters in the early stages of the disease to fully mature, potent TLSs with a GC structure in the late stages. Moreover, the degree of TLS maturation may be related to antitumor immunity.^18^ In this regard, lower recurrence rates have been reported in colorectal and hepatocellular carcinoma patients with mature TLSs.^19,20^

Here, the presence of TLSs was defined based on the aggregation of CD3^+^ T cells and CD20^+^ B cells for the practicality of this clinicopathological investigation, and GC structure formation was used to determine TLS maturity stages. This classification approach is highly reproducible and convenient for clinical practice. The results revealed that TLSs were heterogeneous in UCB, with the highly invasive MIBC having more TLSs than NMIBC. These findings are consistent with those of Koti et al., who studied TLSs in 28 UCB specimens resected by TRUBT in 2017.^7^ It has been implied that TLSs change constantly during the dynamic process of UCB progression. Similar observations have been made in other solid tumors. TLSs have been observed in different tumor progression stages or specimens resected after various treatments, showing a high degree of heterogeneity.^4^ Furthermore, our results showed that in both the NMIBC and MIBC patient groups, TLS presence and the formation of GC structures were accompanied by an increased TIL infiltration, implying the possibility of a more robust antitumor immune response. This finding is consistent with previous observations that TLSs in high-grade plasmacytoid ovarian cancer recruit various lymphocytes while enhancing B cell and CD8^+^ T-cell infiltration, resulting in an antitumor immune response.^21^

Additionally, we discovered that in both the NMIBC and MIBC groups, patients without disease progression had more TLSs and a higher TIL density than those with endpoint events. However, based on the multifactorial Cox analysis results, TLSs and TILs could not be used as independent prognostic indicators for UCB. The multifactorial nature of TLSs could explain this outcome, as the simple typing approach may ignore the composition and role of immune cells in TLSs. However, this does not negate the value of TLSs and TILs in UCB. Various immune cells, including T cells, B cells, DCs, FDCs, and HEVs, are found in TLSs, and their infiltration may mediate antitumor activity. For example, follicular B-cell density in TLSs is positively correlated with more prolonged patient survival in non-small cell lung cancer.^22^ Additionally, a high density of mature DCs in lymphoid aggregates in the tumor stroma was correlated with the intensity of T-cell activation and longer patient survival in primary melanoma.^23^ In breast cancer, HEVs in lymphoid aggregates were associated with longer metastasis-free, disease-free, and overall survival of patients.^24^ Furthermore, the prognostic impact of TLSs may differ depending on their spatial location. In gastric cancer, TLS density in the center of the tumor better reflects the prognosis of patients than that in the infiltrating margins.^25^ In intrahepatic cholangiocarcinoma, a high intratumoral TLS density indicates a good prognosis, whereas a high peritumoral TLS density correlates with a poor prognosis. This difference could be attributed to the heterogeneity of immune cell subpopulations between the TLSs of different locations.^26^ On the other hand, a high TIL infiltration in UCB could be used to identify patients with a favorable prognosis. This is consistent with our univariate Cox analysis results, which revealed that TILs influence the prognosis of patients with NMIBC and MIBC. However, there were differences in TIL infiltration in various molecular subtypes of UCB, with basal tumors having the highest degree of TIL infiltration.^9^ This implies that the use of TILs in clinical prognostic stratification should be supplemented with the molecular characteristics of UCB, which shows a limitation to our study.

The high tumor mutational burden in UCB makes it sensitive to immunotherapy, and particularly, the use of checkpoint inhibitors, such as monoclonal antibodies against programmed cell death 1 (PD-1) and its ligand (PD-L1), has improved OS in metastatic UCB.^27^ Previous research has demonstrated a significant correlation between TLS presence and positive PD-L1 expression in UCB.^28^ A TLS marker, CXCL13, was associated with improved survival in patients with advanced UCB who received immunotherapy.^29^ Additionally, a compositional analysis of the tumor microenvironment in a large cohort of MIBC gene expression datasets showed that TLS signatures were associated with improved survival and response to immunotherapy in MIBC. However, they are yet to be validated in preclinical models.^30^ Overall, while TLSs have great potential as biomarkers of response to immunotherapy in MIBC, their mechanism needs to be supported by further preclinical studies. Although our study did not involve immunotherapy for MIBC, we defined the presence and typing of TLSs in UCB from a clinicopathological standpoint, which is simple, reproducible, and easy to implement clinically, and may provide a foundation for future studies on the role of TLSs in MIBC immunotherapy from a practical clinical perspective.

## Conclusion

Herein, we demonstrated the heterogeneity of TLSs in UCB and found that TLS presence was closely associated with clinical outcomes in patients with UCB. However, the clinical application of TLSs as prognostic markers requires further exploration. Characteristics of TLS presentation and GC formation alone are insufficient indicators of the overall status of TLSs. As a result, using TLS to accurately stratify the clinical prognosis of patients with UCB may be challenging. However, TLSs showed great promise as prognostic stratification markers, and their immune cell composition may be essential in their application as prognostic markers. At the same time, there is a lack of uniform evaluation criteria and specific markers for defining and characterizing TLSs, another area that requires attention for their practical clinical application.

## Supplementary Material

oyad283_suppl_Supplementary_Table_S1

## Data Availability

The data supporting this study’s findings are available from the corresponding author on reasonable request.
