# Supplementary material for: Presence, Subtypes, and Prognostic Significance of Tertiary Lymphoid Structures in Urothelial Carcinoma of the Bladder
Source: Oncologist. 2023 Oct 24;29(2):e248–58. doi: 10.1093/oncolo/oyad283 (PMC10836299; doi:10.1093/oncolo/oyad283)
Supplement: oyad283_suppl_Supplementary_Table_S1 [file oyad283_suppl_supplementary_table_s1.docx]

| **Supplementary Table1**：**Clinicopathological characteristics of NMIBC and MIBC.** | | | |
| --- | --- | --- | --- |
| **Clinicopathological** | All | NMIBC | MIBC |
| **characteristics** | (N=580) | (N=313) | (N=267) |
| **Age /years** |  |  |  |
| Median/range | 68/30-93 | 68/30-90 | 68/36-93 |
| Mean±SD | 68.2±10.2 | 68.1±10.1 | 68.3±10.3 |
| **Gender** |  |  |  |
| Male | 480 | 253 | 227 |
| Female | 100 | 60 | 40 |
| **Tumor size/cm** |  |  |  |
| Median/range | 3/0.2-15 | 3/0.2-10 | 4/1-15 |
| Mean±SD | 3.6±2.0 | 3.0±1.7 | 4.2±2.1 |
| **Tumor multiplicity** |  |  |  |
| Single | 490 | 261 | 229 |
| Multiple | 90 | 52 | 38 |
| **Histological subtype** |  |  |  |
| IUC | 486 | 279 | 207 |
| IUC with divergent differentiation | 58 | 21 | 37 |
| IUC variants | 36 | 13 | 23 |
| **pT** |  |  |  |
| 1 | 313 | 313 | / |
| 2 | 142 | / | 142 |
| 3 | 95 | / | 95 |
| 4 | 30 | / | 30 |
| **Lymph node metastasis** |  |  |  |
| Positive | 52 | 3 | 49 |
| Negative | 196 | 73 | 123 |
| Unknown | 332 | 237 | 95 |
| **Perineural Invasion** |  |  |  |
| Positive | 80 | 6 | 74 |
| Negative | 500 | 307 | 193 |
| **LVI** |  |  |  |
| Positive | 115 | 14 | 101 |
| Negative | 465 | 299 | 166 |
| **Ki-67** |  |  |  |
| Median/range | 40/1-90 | 40/1-90 | 40/4-90 |
| Mean ± SD | 42.5±22.5 | 36.8±23.1 | 45.9±21.5 |
| Unknown | 308 | 214 | 94 |
| **Treatment method** |  |  |  |
| TURBT | 11 | 11 | 0 |
| TURBT+IC | 86 | 78 | 8 |
| TURBT+BCG | 144 | 126 | 18 |
| PC+BCG | 10 | 4 | 6 |
| RCT | 75 | 20 | 55 |
| RCT+RT | 3 | 0 | 3 |
| RCT+CT | 6 | 0 | 6 |
| RCT+PLAD | 203 | 74 | 129 |
| RCT+PLAD+RT | 12 | 0 | 12 |
| RCT+PLAD+CT | 27 | 0 | 27 |
| RCT+PLAD+CRT | 3 | 0 | 3 |
| **Resection status** |  |  |  |
| R0 | 512 | 264 | 248 |
| R1 | 66 | 49 | 17 |
| R2 | 2 | 0 | 2 |
| **Endpoint events** |  |  |  |
| With | 191 | 85 | 106 |
| Without | 379 | 225 | 154 |
| Unknown | 10 | 3 | 7 |
| Abbreviations: NMIBC: non-muscle invasive bladder cancer; MIBC: muscle invasive bladder cancer; IUC: Invasive urothelial carcinoma; TILs: tumor-infiltrating lymphocytes; LVI: lymphovascular invasion; TLSs: tertiary lymphoid structures; nGC TLSs: non-germinal center TLSs; GC TLSs: germinal center TLSs; TURBT: transurethral resection of bladder tumor; IC: intravesical chemotherapy; BCG: BCG intravesical immunotherapy; PC: partial cystectomy; RCT: radical cystectomy; RT: radiotherapy; CT: chemotherapy; CRT: chemoradiotherapy; PLAD: pelvic lymph node dissection; SD: standard deviation. | | | |
